# Biomechanical Comparison of the New-Generation Implant Designed for the Fixation of Patella Fractures with the Tension Band Method

**DOI:** 10.3390/medicina61060952

**Published:** 2025-05-22

**Authors:** Ahmet Ülker, Ahmet Burak Satılmış, Zafer Uzunay, Tolgahan Cengiz, Abdurrahim Temiz, Mustafa Yaşar, Tansel Mutlu, Uygar Daşar

**Affiliations:** 1 Department of Orthopedics and Traumatology, Mersin University, Mersin 33110, Turkey; drahmetulker@gmail.com; 2 Department of Orthopedics and Traumatology, Taşköprü State Hospital, Kastamonu 37400, Turkey; tolgahancengiz@hotmail.com; 3 Department of Orthopedics and Traumatology, Medicalpark Adana Hospital, Adana 01060, Turkey; dr.zuzunay@gmail.com; 4 Department of Industrial Design Engineering, Karabük University, Karabük 78050, Turkey; abdurrahimtemiz@karabuk.edu.tr (A.T.); myasar@karabuk.edu.tr (M.Y.); 5 Department of Orthopedics and Traumatology, Medicalpark Gebze Hospital, Kocaeli 41400, Turkey; tanselmutlu@yahoo.com; 6 Department of Orthopedics and Traumatology, Karabük University, Karabük 78050, Turkey; udasar@yahoo.com

**Keywords:** patella fractures, fracture fixation, tension band wiring, biomechanics, implant

## Abstract

*Background and Objectives*: This study compares the biomechanical performance of a new-generation implant designed for patella fracture fixation with the traditional tension band method. Its goal is to assess fracture fixation’s stability and the new implant’s potential advantages in reducing complications such as skin irritation, pain, and implant failure. *Materials and Methods*: In this experimental study, 20 calf patellae were divided into two groups. The first group was treated with the traditional tension band method, while the second group received the new-generation implant, designed using finite element analysis (FEA) for optimization. Both groups underwent biomechanical testing with axial forces at a 45° flexion angle to simulate real-life load conditions. The maximum forces at which mechanical insufficiency occurred were recorded. Data were analyzed using SPSS for statistical comparison. *Results*: Finite element analysis revealed that the new-generation implant provided better fracture line stability than the tension band method under applied forces. In the biomechanical tests, the maximum force at which mechanical insufficiency occurred was significantly higher in the new-generation implant group (1130 ± 222 N) compared to the tension band method group (680.5 ± 185.4 N), with a statistically significant difference (*p* = 0.008). The new implant demonstrated superior fixation, with better resistance to distraction forces. *Conclusions*: The new-generation implant offers enhanced biomechanical stability compared to the traditional tension band method, particularly regarding fixation strength under applied forces. This study supports the potential of the new implant to improve fixation stability and reduce common complications associated with patella fracture surgeries. Further testing in more extensive human cadaver studies is recommended to confirm these findings and assess long-term clinical outcomes.

## 1. Introduction

The patella is located on the anterior surface of the knee joint, and fractures occur more frequently due to direct trauma. The incidence of patella fractures is 1.2–6.1 per 100,000, constituting approximately 3.5% of lower-extremity fractures [1,2,3]. They are common in men aged 20–50 [4]. Many patella fracture fixation methods have been suggested in the literature. They include various screw designs, K-wires, compressive pins, stainless steel wires, braided sutures, locking and non-locking plates, external fixators, and multiple combinations of these implants [5,6]. Fixation with cannulated screws, in particular, has the potential to provide better compression at the fracture line. At the same time, plate–screw systems are preferred in cases with multi-fragmented or osteoporotic bone structures. However, each of these methods has its advantages and complications. In the literature, the biomechanical performances of these techniques have been compared, but there is still no consensus on an ideal method. Therefore, developing new-generation implants and their comparative evaluation with the existing systems are essential for clinical applications. In our country and around the world, the tension band method is most commonly used for fracture fixation in treating patella fractures. The tension band method includes two parallel K-wires perpendicular to the fracture line and one stainless steel wire surrounding the K-wires, which looks like the number eight, on the anterior surface of the patella.

The patella is located just under the skin and does not have muscle, fat, or soft tissue support, as in other body parts. The main complications are implant fracture, wire penetration into the skin, and anterior knee pain [5]. Complications have been reported between 18% and 50% in patients with transverse patella fractures treated with the tension band method [6,7]. These complications have led surgeons to seek new surgical techniques and implants. In our study, we designed a new-generation implant considering the results of old implants, which we expect to minimize the complications seen in the current fixation methods. This designed implant was optimized and manufactured with finite element analysis. The functionality of the implant was compared biomechanically with the classical tension band method in the transverse fracture model created in the calf patella bone. We aim to evaluate the data we obtained as a result of this study in light of the research information and to determine the advantages and disadvantages of the new-generation implant we developed.

## 2. Materials and Methods

In this experimental study, a power analysis was performed, and 20 cadavers with intact knee joint integrity were obtained. All samples were frozen in closed plastic containers within 8 h after death. The samples were thawed at room temperature on the test day and kept moist with physiological saline solution throughout the test procedure. The calf knees were randomly divided into two groups. The patellar tendon integrity was intact, but since there was no quadriceps tendon, a transverse tunnel was opened with a 5 mm drill to simulate the patella proximally, and one piece of 5 mm stainless steel wire was passed. The patella was cut with a saw from the broadest part, creating a transverse patella fracture.

**Figure 1:**
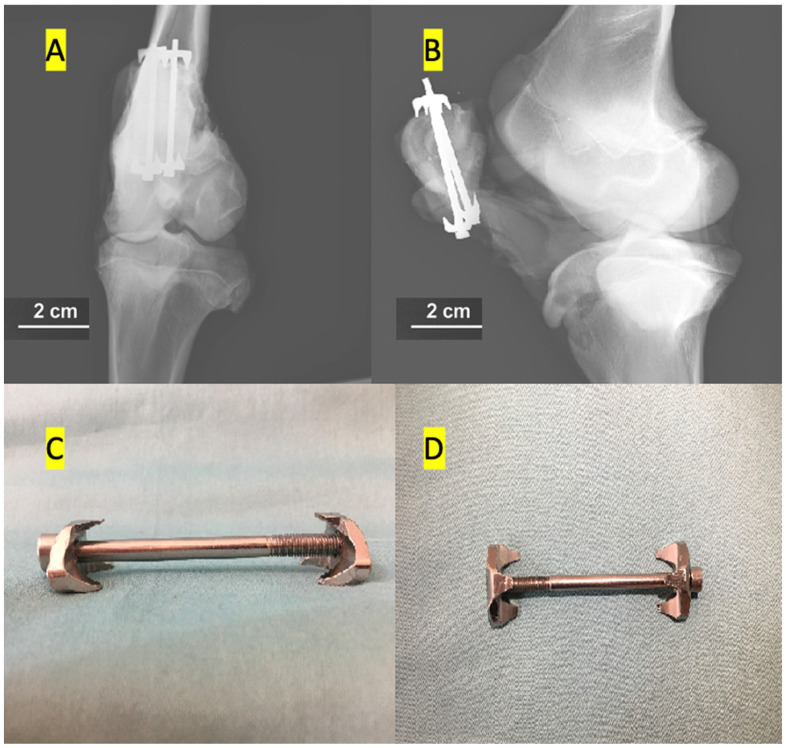
Radiographic and macroscopic views of the newly designed implant for patella fracture fixation. (**A**) Anteroposterior X-ray of the calf knee after fixation with the new-generation implant. (**B**) Lateral X-ray of the calf knee demonstrating the implant in situ. (**C**) Lateral view of the newly designed implant. (**D**) The top view of the implant shows a bilateral hook configuration.

The classically accepted tension band method was applied to the first group. Briefly, 2 mm K wires and 1.25 mm cerclage wires were used for fracture fixation. The new-generation implant fixation method was used in the second group. The implant was designed in the ANSYS R19.1 (Canonsburg, PA, USA) program. The implant consists of 3 parts: 2 hooks and 1 screw. A half-threaded cannulated screw (outer diameter of 4 mm, 207.642, DePuy Synthes, Paoli, PA, USA) was simulated as the screw. While there is a groove in the hook to be attached to the distal part of the screw, there is no groove in the hook attached to the proximal part of the screw (Figure 1). Thus, as the screw is tightened, it creates compression on the fracture line. Biomechanical measurement analysis was performed for each group after the implants were applied. This study was conducted at the Karabük University Iron and Steel Institute Margem Biomechanics Laboratory using a testing device (MTS Landmark Testing Solutions, Eden Prairie, MN, USA) that can apply axial forces to biomaterials and measure biomechanical changes in materials.

The literature shows that the maximum load angles on the patella are between 45° and 60°. The loading angle was chosen to simulate the load experienced in the standing movement just before sitting [8]. A fixed angle of 45° was preferred in this study. A special apparatus was designed and manufactured to place the samples in the device and to keep the knee joint fixed at 45° flexion. Holes were drilled on the femur and tibia and fixed to the specially designed apparatus with the help of steel wires. The knee was fixed at 45° flexion. The metal piece attached to the end of the 5 mm steel wire passing through the upper pole of the patella was placed in the upper mouth of the device. A tensile force was applied at a speed of 2 mm/min. The forces detected immediately after the 2 mm separation in the fracture line were accepted as causing fixation insufficiency. The maximum forces at which the test was terminated were recorded. This study was conducted with approval from the university’s Non-Interventional Clinical Research Ethics Committee (number: 77192459-050.99/514054 Date: 15 January 2018).

### Statistical Analysis

The data were evaluated using the Statistical Package for the Social Sciences (SPSS) for Windows version 20.0. Descriptive statistics for categorical variables were presented as numbers and percentages, and for numerical variables, as mean ± standard deviation and minimum–maximum values. In the analysis of numerical data, compliance with normal distribution was examined with the “Kolmogorov–Smirnov” and “Shapiro–Wilk” tests, and since the only numerical variable, maxkNw, showed normal distribution, the mean difference between the two groups was examined with the “Independent samples” test. The data were reviewed at a 95% confidence level, and if the *p*-value was of less than 0.05, the tests were considered significant.

## 3. Results

**Figure 2:**
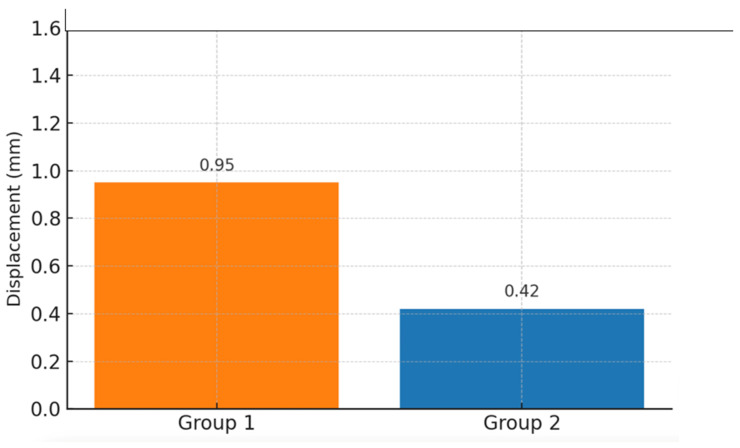
Comparison of the separation amounts at the fracture line at 0° force angle. The separation amounts measured for Group 1 (tension band) and Group 2 (new-generation implant) are 0.95 mm and 0.42 mm, respectively.

**Figure 3:**
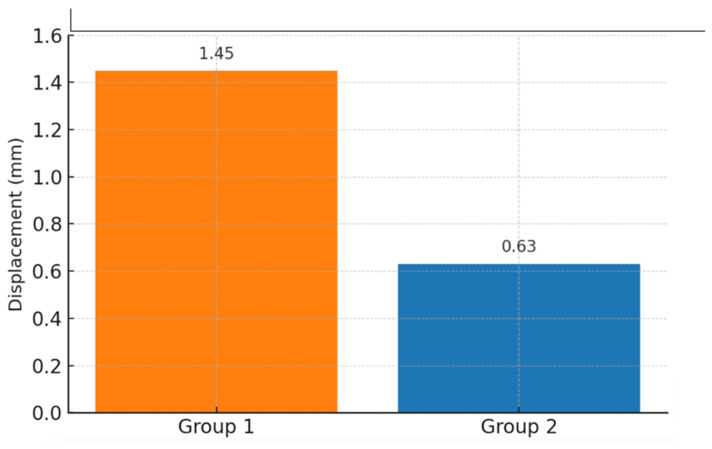
Comparison of the separation amounts at the fracture line at a force angle of 45°. A separation of 1.45 mm was observed for Group 1 and 0.63 mm for Group 2 (Group 1: tension band, Group 2: new-generation implant).

**Figure 4:**
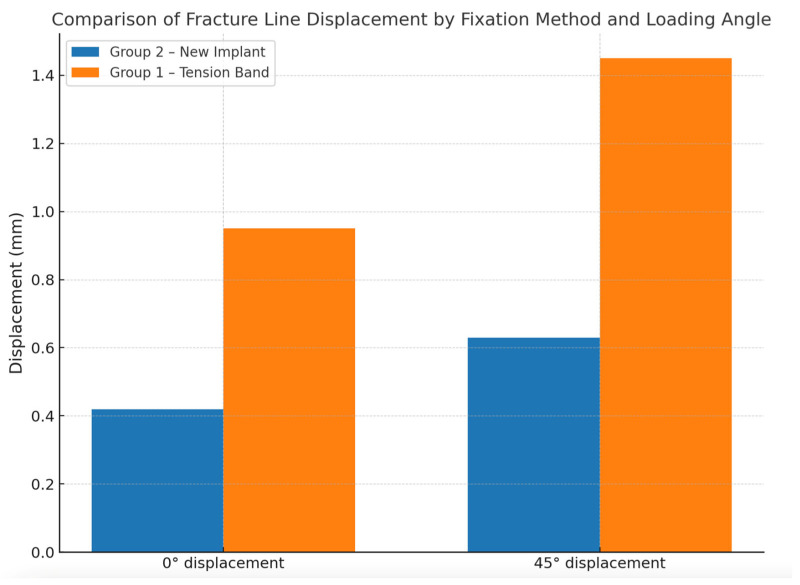
The finite element analysis results compare the new-generation implant with the classical tension band method when an 850 N force is applied. *X* axis: applied method (Group 1: tension band, Group 2: new-generation implant), *Y* axis: amount of separation at the fracture line (mm).

A finite element analysis of both groups was performed. The total deformation of the system was recorded for the case where an 850 N force was applied at an angle of 45° (Figure 2). The maximum separation was approximately 0.63 mm on the patella’s anterior side. The separation at the fracture line was recorded for the case where an 850 N force was applied at an angle of 0° (Figure 3). The maximum separation was approximately 0.42 mm on the patella’s anterior side. In the finite element analysis where an 850 N distraction force was applied, the results of the new-generation implant were better than those of the tension band method in Group 1 (Figure 4).

**Figure 5:**
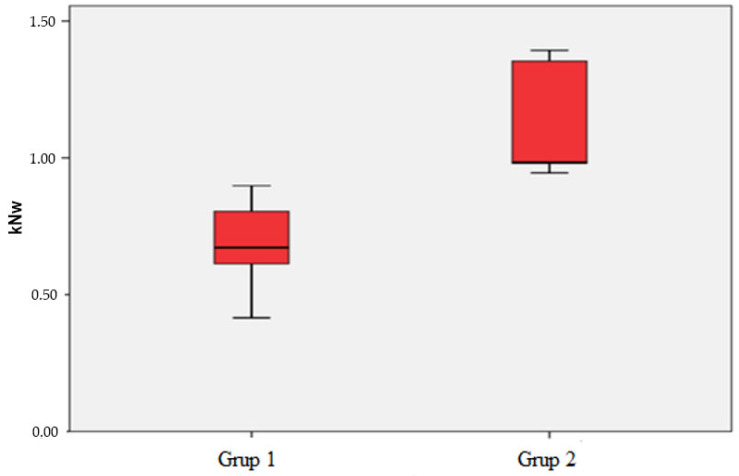
Force distributions where mechanical failure occurs.

The biomechanical analysis recorded the maximum forces at which mechanical insufficiency occurred due to distraction after fracture fixation (Table 1). The device applied distraction at a 2 mm/min speed. The 2 mm separation in the fracture line was measured, and the test was terminated accordingly. In the first group, the average of the forces at which mechanical insufficiency occurred was 680.5 ± 185.4 N (415–898). In the second group, the same values were found to be 1130 ± 222 N (945–1392) (Figure 5). Since *p* = 0.008 (<0.05) was found, a statistically significant difference was detected between the means of maximum force values according to the groups. The average maximum force value of Group 2 was significantly higher than that of Group 1 (*p* < 0.05). No patellar tendon avulsion was observed during mechanical testing in any specimens; the tendon deformation visible in the lateral X-ray image resulted from the incision made during specimen preparation.

## 4. Discussion

Patella fractures have been treated with various methods over the past century. Although surgical indications have been determined with clear criteria, there still needs to be a consensus on the surgical technique. Non-comminuted transverse fractures constitute approximately half of patella fractures [9], and are most commonly treated with the tension band method [10]. Although the tension band method is the most widely used fixation technique for patella fracture fixation today, poor clinical results have been reported in up to 55% of cases [11]. In addition, the implant removal rate is approximately 40% due to skin irritation and pain in the anterior knee [12,13]. Rotation of the ends of the K-wires, loosening of the cerclage wire, and displacement are the main problems. When considering the literature, publications on patella fractures generally include new implant searches and their biomechanical studies [8,14,15,16,17]. In our study, we designed a new-generation implant to improve mechanical stability and overcome the shortcomings of the traditional technique. Finite element analysis and biomechanical testing showed that the new implant demonstrated significantly less displacement at the fracture line under 850 N axial load and substantially higher resistance to distraction forces than the classical tension band method (*p* = 0.008). The new implant was designed considering that the implant should not be between the anterior surface of the patella and the skin to prevent skin irritation and pain that may develop in case of common complications, such as skin irritation, foreign body sensation, and minor traumas from the front of the knee.

In the surgical treatment of osteoporotic bone fractures, hook-shaped devices that can be attached to screws were added due to the inadequacy of fixation techniques performed only with screws. Since the triple hook structure in the implant we designed will be fixed to the outer cortex of the patella, rotational movement of the implant is not expected. While there is no groove in the hook that fits the screw head, there is a groove in the hook in the distal part. Thus, the desired compression can be achieved in the fracture line as the screw is tightened. In addition, since the hooks will attach cortical bone structure to the bone structure, stability will be better in osteoporotic patients. Since the implant will take up more space than the tension band method, it may cause more damage to the patellar and quadriceps tendons. However, if the implant is sized according to the size of the patella or modified with clinical experience, its complications may be fewer. In a cadaver study, no significant difference was found between the groups in a biomechanical study comparing the tension band method with the cannulated screw and another fixation method in which a bolt and nut were added to the screw tip [18]. We believe that the implant we developed will contribute more to stability because it can be clamped with screws and has extra hooks.

Before starting this experimental study, the aim was to produce the optimum implant by performing finite element model analysis. The implant design was modified according to the analysis of the finite element model. The finite element analysis of the new-generation implant was performed by taking the study of Chang et al. as a reference [19]. After obtaining similar results, implant production and biomechanical testing were started. Both the patellofemoral contact area configuration with knee flexion and the biconvex shape of the patella create bending moments on the anterior surface of the patella. In the literature, the value at which bending moments reach their maximum has been reported at 45° flexion of the knee [20]. We also conducted our study in a position where the knee was fixed at 45° flexion.

In biomechanical studies on patella fractures, cadaver patellas are used to provide more accurate results [21]. Due to difficulties obtaining cadavers, publications use the calf patella model [22]. When evaluating the results, it should be remembered that the calf patella used contains some differences from the human patella and may affect the results. The first of these is that it is unknown to what extent the cortical and cancellous parts of the calf patella differ from the human patella and whether the fixation methods used affect its stabilization. The calf patella is larger than the human patella, and the increase in the materials’ dimensions may biomechanically affect its stabilization and resistance to distraction forces.

Çekin et al. compared three different fixation methods biomechanically in 30 calf patellas where transverse fractures were created. They used the tension band method, a 4.5 mm headless compression screw, and the 4.5 mm headless compression screw and tension band method as fixation methods [23]. In the experiment where distraction forces were simulated in full extension, the technique created with a tension band via a 4.5 mm headless compression screw provided the strongest fixation. The tension band method performed with K-wires was reported as the weakest fixation. In our study, unlike Çekin et al., we preferred calf knees with intact joint integrity. In addition, while applying distraction force to the patella, we fixed the knee joint in a 45° flexion position, and the patellofemoral joint surfaces were in contact. Therefore, it should be considered that there may be vectorial differences between the forces applied to the fracture line during distraction, and the results of both studies should be compared accordingly.

In another biomechanical study using the calf knee, Gürbüz et al. compared three techniques in transverse patella fractures [24]. Among the groups subjected to the tension band method, malleolar screw fixation, and tension band method via the Herbert screw, the most rigid fixation resistant to distraction forces was found to be the technique applied with the malleolar screw fixation and tension band method via the Herbert screw. The tension band method was found to be the weakest fixation method. The samples contained intact calf knee joints, and the technical details used to perform the test are similar to those in our study.

Dargel et al. biomechanically compared three different fixation methods in 106 calf knees, in which they created transverse patella fractures [22]. The tests were repeated in positions where the knee joint was fixed at 0° and 45°. Fracture fixation was performed with two cannulated screws in the first group, with the tension band method in the second group, and with three mini-screw fragment fixation systems (FFS-large, OrthofixTM, Italy) in the third group. While no significant difference was found between the groups in the tests performed at 0° and 45° angles, the forces causing separation in the fracture line in the test performed at 45° were found to be smaller than those when the test was performed at 0°. Again, Schnabel et al. biomechanically compared the tension band method with the staple fixation technique. Unlike us, the authors preferred the fatigue test over the increase in the traction force in their experimental study. They simulated the active joint movement required to avoid losing the range of motion in the early period after the surgical treatment of patella fractures. They found the staple fixation technique to be superior [8].

One of the most striking aspects of this study is that a new-generation implant, which has no previous equivalent in the literature, has been evaluated both with its optimized design based on finite element analysis and with biomechanical comparisons. Although many methods have been proposed in the recent literature for treating patellar fractures, there is still no consensus on an ideal fixation technique. This study represents an innovative approach to solving the frequently encountered complications of the classical tension band method. The fact that the implant is not located on the anterior surface has the potential to reduce complications such as skin irritation and wire migration. In addition, the findings obtained in both experimental and numerical analyses show that the implant exhibits superior performance in terms of mechanical stability. This design can provide an alternative to the stability problems frequently encountered in treating patellar fractures and can shed light on future clinical studies. The systematic execution of the modeling, design, production, and testing processes is a strength that distinguishes this study from its peers. Therefore, this study significantly contributes to the literature regarding innovative biomechanical design and clinical applicability.

This study has several limitations. First, biomechanical analyses were performed only at 0° and 45° angles; however, in the literature, the angles at which the patellofemoral joint is exposed to maximum loading are usually between 45° and 60°. Therefore, analyses at angles such as 30°, 60°, and 75° will provide more comprehensive information in terms of the clinical applicability of the implant. This study was performed in vitro, and the surrounding soft tissues (muscle, tendon, ligament tissue) were not modeled; this caused the real biomechanical conditions not to be fully reflected. In addition, the quadriceps muscle effect was simulated using only a fixed tensile force; different force variations were not evaluated. The model used, of the calf patella, is incompatible with the human patella in terms of anatomical structure and bone density. These differences may affect the implant’s stability and limit the direct generalization of the results to human anatomy. In addition, this study is limited to a simple transverse fracture model; more complex fracture types were not evaluated. Finally, the sample size is relatively limited, and bone mineral density was not measured objectively. This situation limits the evaluation of implant success, especially in osteoporotic bones. For these reasons, further studies on human cadavers with larger sample groups and variable angles and forces are needed.

## 5. Conclusions

The new-generation implant, which will provide a more stable fixation in the surgical treatment of patella fractures and minimize the complications related to patella fractures, was compared biomechanically with the tension band method in a calf patella model. The finite element analysis performed before the experiment determined that the new-generation implant provided better stability than the tension band method at the fracture line. As a result of the biomechanical experiment, it was seen that the new-generation implant provided a more stable fixation than the tension band method. This study was conducted using the pioneering design of the implant. The implant should be tested in larger groups in the future, and human cadaver studies should confirm the results.

## Figures and Tables

**Table 1:** Maximum force (N) analysis by groups.

Group	Number of Subjects	Median Value (Standard Deviation)	Min–Max Values
Group 1	10	680.52 (185.4)	415–898
Group 2	10	1130.7 (222.1)	945–1392
Total	20	905.6 (305.8)	415–1329

## Data Availability

All data can be transmitted to this journal if requested.
